# Supplementary figures and images for: Effectiveness of anti-vascular endothelial growth factors in neovascular age-related macular degeneration and variables associated with visual acuity outcomes: Results from the EAGLE study
Source: PLoS One. 2021 Sep 1;16(9):e0256461. doi: 10.1371/journal.pone.0256461 (PMC8409622; doi:10.1371/journal.pone.0256461)

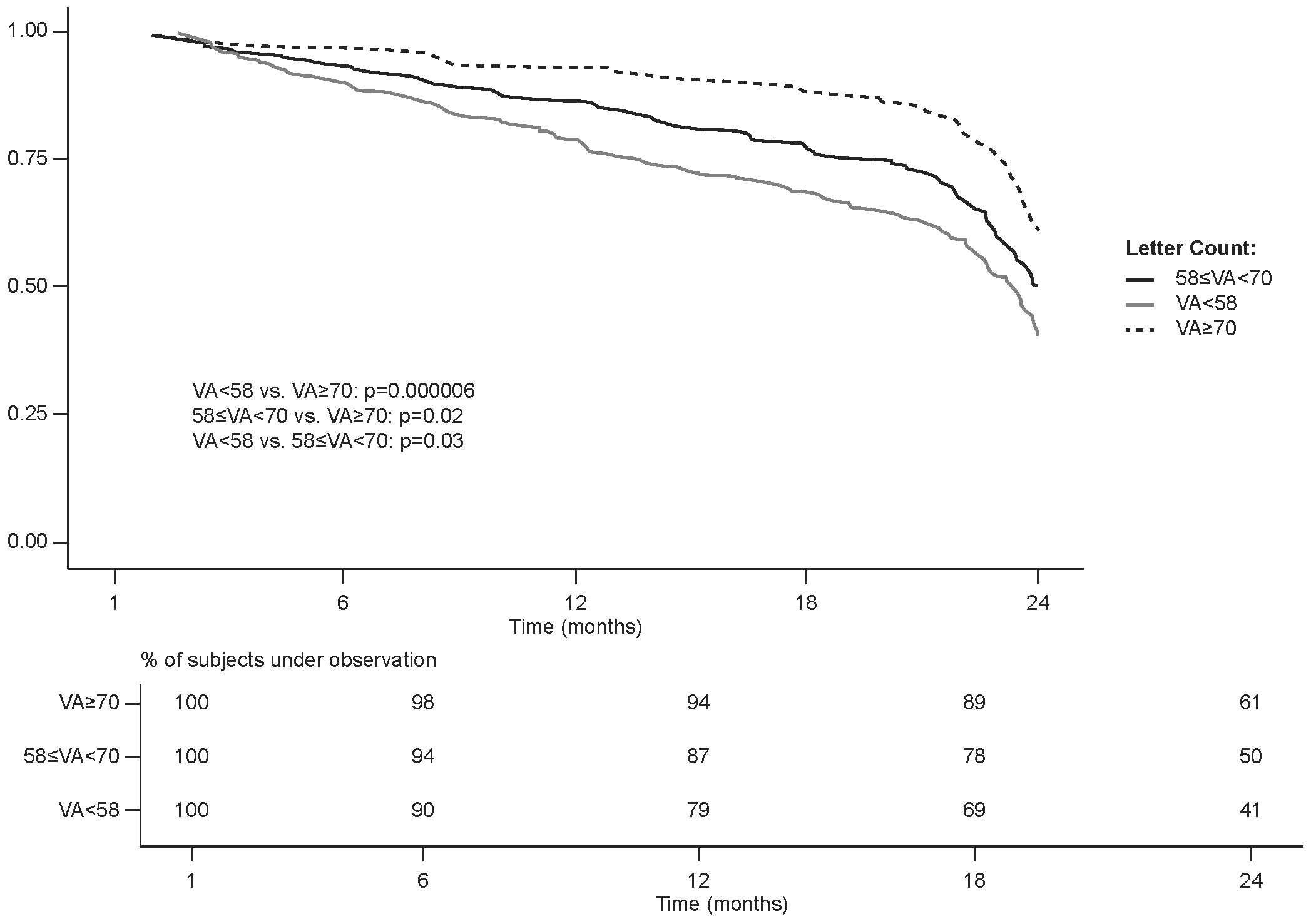

Supplement: S1 Fig — EA set: All patients in the OE who had a baseline and at least one post-baseline assessment of VA. At Month 12, 94% of patients with baseline VA ≥70 ETDRS letters, 87% of patients with baseline VA ≥58 and <70 ETDRS and 79% of patients with baseline VA <58 were under observation. At Month 24, 61%, 50% and 41% of patients with baseline VA ≥70, ≥58 and <70 ETDRS and <58 ETDRS letters, respectively, were still under observation. EA, effectiveness analysis; ETDRS, early treatment diabetic retinopathy study; VA, visual acuity. According to the application of Bonferroni correction due to multiple comparisons the threshold of significance was set at 0.02. (TIF) [file pone.0256461.s001.tif]

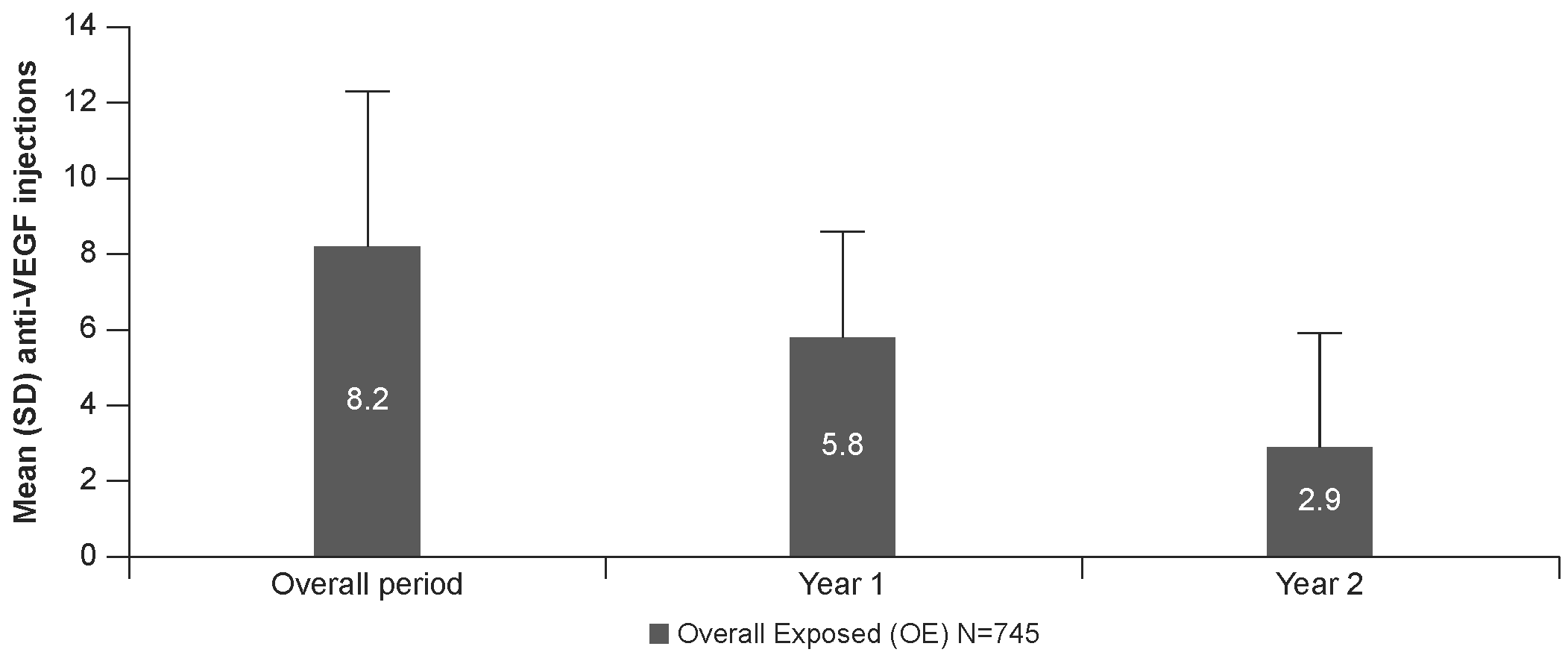

Supplement: S2 Fig — OE population: All enrolled patients who had at least one anti-VEGF injection; Mean (SD) number of anti-VEGF injections received by OE populations during Year 1 (until Month 12), Year 2 (Months 13–24) and overall period are presented. n, number of patients; OE, overall exposed; SD, standard deviation. (TIF) [file pone.0256461.s002.tif]

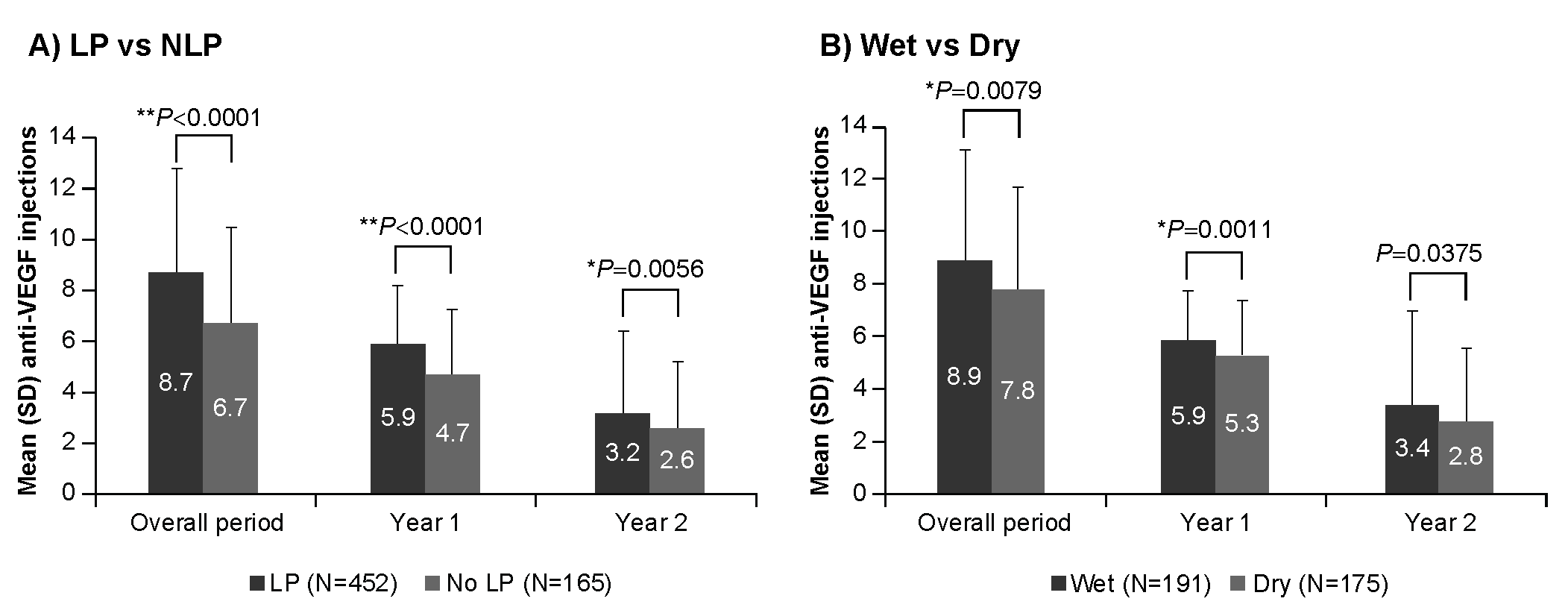

Supplement: S3 Fig — Mean (SD) number of injections in patients during Year 1, 2 and overall period are presented based on (A) those completing LP or NLP; and (B) in patients classified as wet or dry based on investigators discretion at the end of LP. LP, loading phase; NLP, no loading phase; SD, standard deviation. (TIF) [file pone.0256461.s003.tif]

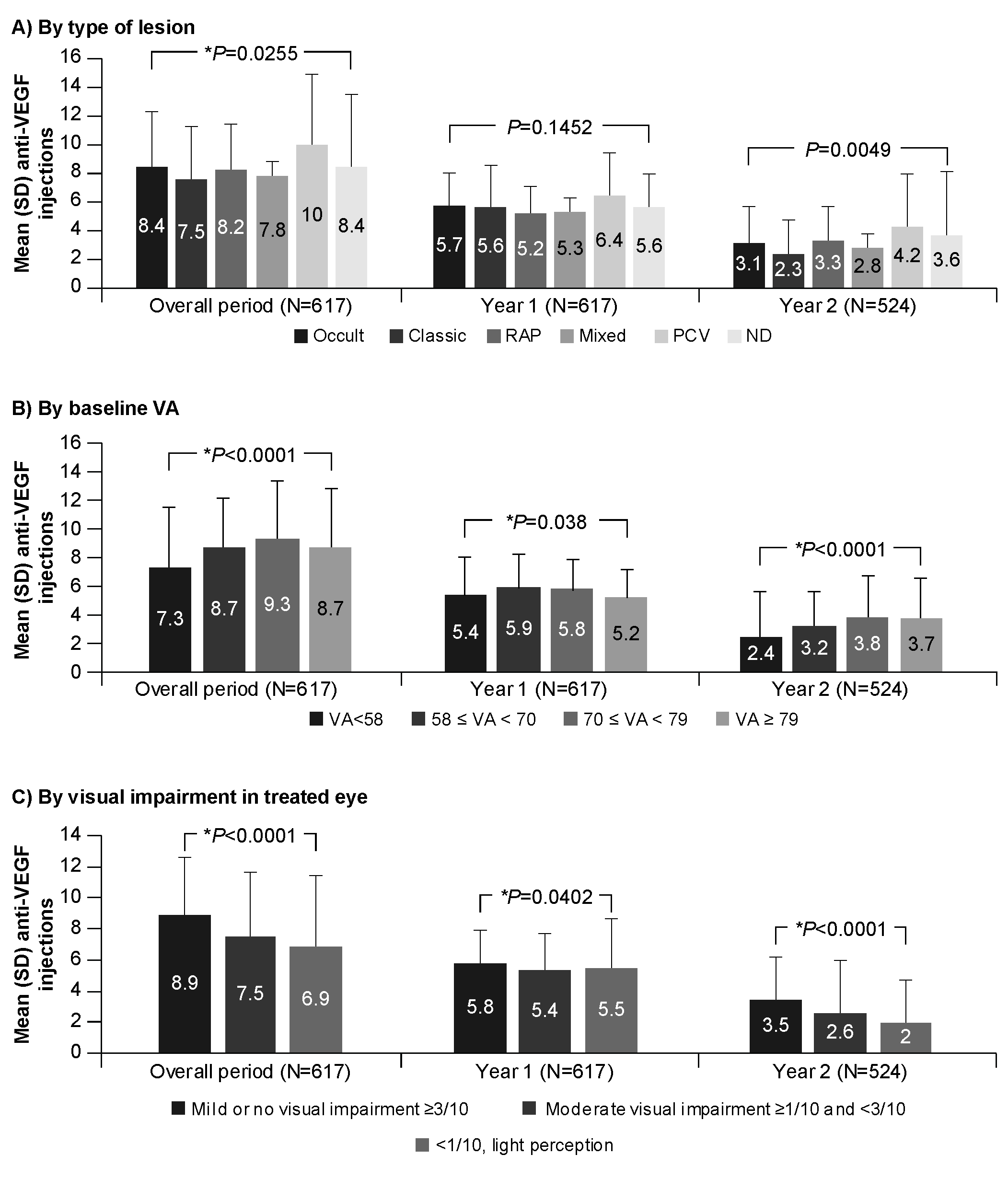

Supplement: S4 Fig — Mean (SD) annualized anti-VEGF injections in EA population during the Year 1, 2 and overall period were evaluated based on baseline ocular characteristics (A) type of lesion, (B) baseline VA, and (C) visual impairment in the treated eye. EA, effectiveness analysis; SD, standard deviation; VEGF, vascular endothelial growth factor. (TIF) [file pone.0256461.s004.tif]
