# Supplementary material for: Effectiveness of anti-vascular endothelial growth factors in neovascular age-related macular degeneration and variables associated with visual acuity outcomes: Results from the EAGLE study
Source: PLoS One. 2021 Sep 1;16(9):e0256461. doi: 10.1371/journal.pone.0256461 (PMC8409622; doi:10.1371/journal.pone.0256461)
Supplement: S1 Table — (DOCX) [file pone.0256461.s005.docx]

**Table S1: List of EAGLE study investigational sites and related ethics committees**

| **Site Number** | **Site Name** | **Ethics Committee** |
| --- | --- | --- |
| 1 | S.C. di Oculistica, Università degli Studi, Presidio Ospedali Riuniti di Foggia, Az. Osp. Ospedali-Università OO.RR. Foggia Via Luigi Pinto, Foggia | Comitato Etico Dell'azienda Ospedaliero-Universitaria Ospedali Riuniti Di  Foggia  Via Luigi Pinto,  71100 Foggia (Fg) |
| 2 | U.O.C. di Oculistica, Università degli Studi di Palermo, Az. Ospedaliera Universitaria Policlinico P. Giaccone  Via Liborio Giuffrè, 13, Palermo | Comitato Etico Palermo 1 C/O Aou Policlinico P. Giaccone Di Palermo  Via Del Vespro, 127  90127 Palermo (Pa) |
| 3 | U.O.D. Oculistica, Università La Sapienza, Azienda Ospedaliera Sant'Andrea  Via di Grottarossa, 1035/1039, Roma | Comitato Etico Dell'universita' Sapienza  Viale Del Policlinico, 155  00161 roma (rm) |
| 4 | S.C.O. di Oculistica, Azienda Ospedaliero-Universitaria Maggiore della Carità  Corso Mazzini, 18, Novara | Comitato Etico Interaiendale Dell'azienda Ospedaliera Universitaria  Maggiore Della Carita' Di Novara  Corso Mazzini 18  28100 Novara (No) |
| 5 | Clinica Oculistica – DINOGMI, Ospedale Policlinico San Martino IRCCS  Largo Rosanna Benzi, 10, Genova | Comitato Etico Regionale Della Liguria C/O Irccs Aou San Martino - Ist  Istituto Nazionale Per La Ricerca Sul Cancro  Largo Rosanna Benzi, 10  16132 Genova (Ge) |
| 6 | U.O. Oculistica Universitaria, Presidio Ospedaliero di Cisanello, Az. Ospedaliero Universitaria Pisana  Via Paradisa, 2, Pisa | Comitato Regione Toscana - Area Vasta Nord Ovest C/O Azienda Ospedaliero  Universitaria Pisana Di Pisa  Via Roma, 67  56126 Pisa (Pi) |
| 7 | U.O.S.D. Patologie Retiniche Unità Patologie Oculari Croniche Degenerative, Università degli Studi Tor Vergata, Fondazione Policlinico Tor Vergata  Viale Oxford, 81, Roma | Comitato Etico Indipendente Presso La Fondazione Ptv Policlinico Tor  Vergata Di Roma  Viale Oxford, 81  00133 Roma (Rm) |
| 8 | S.O.C. Clinica Oculistica, Università degli Studi, Presidio Ospedaliero Santa Maria della Misericordia di Udine, Azienda Sanitaria Universitaria Integrata di Udine  P.le S.M. della Misericordia, 15, Udine | Comitato Etico Unico Regionale (Ceur) C/O Direzione Scientifica Del Centro Di Riferimento  Oncologico Di Aviano  Via Gallini 2  33081 Aviano (Pn) |
| 9 | Divisione di Oftalmologia, Università degli Studi, Presidio Ospedaliero Gaspare Rodolico, Az. Osp. Univ. Policlinico-Vittorio Emanuele  Via Santa Sofia, 78, Catania | Comitato Etico Catania 1 - c/o a.o.u. Policlinico-Vittorio Emanuele Di  Catania  Via Santa Sofia, 78  95123 Catania (Ct) |
| 10 | Clinica Oftalmologica, Università G. D'Annunzio, P.O. Ospedale Clinicizzato SS. Annunziata Colle Dell'Ara, ASL n. 2 - Lanciano Vasto Chieti  Via dei Vestini, 5, Chieti | Comitato Etico Per Le Province Di Chieti E Pescara  Via Dei Vestini, 29b  66100 Chieti Scalo (Ch) |
| 11 | S.O.D. Oculistica - Ottica Fisiopatologica, Università degli Studi di Firenze, Azienda Ospedaliero-Universitaria Careggi  Largo Giovanni Alessandro Brambilla, 3, Firenze | Comitato Etico Regione Toscana - Area Vasta Centro C/O Azienda  Ospedaliero-Universitaria Careggi Di Firenze  Largo Giovanni Alessandro Brambilla, 3  50134 Firenze (Fi) |
| 12 | U.O.C di Oculistica, Università degli Studi, Azienda Ospedaliera Luigi Sacco Polo Universitario, ASST Fatebenefratelli Sacco  Via Giovanni Battista Grassi, 74, Milano | Comitato Etico Interaziendale Milano Area 1 C/O Asst Sacco Fatebenefratelli  Via Giovanni Battista Grassi, 74  20157 Milano (Mi) |
| 13 | U.O. di Oculistica, IRCCS Ospedale San Raffaele  Via Olgettina, 60, Milano | Comitato Etico Dell'irccs Ospedale San Raffaele Di Milano  Via Olgettina, 60  20132 Milano (Mi) |
| 14 | U.O.C. di Oculistica Fondazione IRCCS Ca' Granda Ospedale Maggiore Policlinico, Università degli Studi,Via Manfredo Fanti, 6, Milano | Comitato Etico Milano Area 2 C/O Fondazione Irccs Ca' Granda Ospedale  Maggiore Policlinico - Palazzo Uffici - 2° Piano  Via Francesco Sforza, 28  20122 Milano (Mi) |
| 15 | U.O. di Oftalmologia Clinica Oculistica, Azienda Ospedaliero-Universitaria di Sassari  Viale San Pietro, 43, Sassari | Comitato Etico Indipendente Dell'azienda Ospedaliera Universitaria Di  Cagliari  Via Ospedale, 54  09124 Cagliari (Ca) |
| 16 | U.O.S. Centro Maculopatie Clinica Oculistica, Università La Sapienza, A.O. Policlinico Umberto I  Viale del Policlinico, 155, Roma | Comitato Etico Dell'universita' Sapienza  Viale Del Policlinico, 155  00161 Roma (Rm) |
| 17 | U.O.S. Retina Medica, Presidio Ospedaliero Britannico, IRCCS Fondazione G.B. Bietti  Via di Santo Stefano Rotondo, Roma | Comitato Etico Centrale Irccs Lazio - Sezione Ifo-Bietti C/O Irccs Istituti  Fisioterapici Ospitalieri Di Roma  Via Elio Chianesi, 53  00144 Roma (Rm) |
| 18 | U.O. Oftalmologia Universitaria, Università degli Studi, Az. Osp. Univ. Consorziale Policlinico di Bari  Piazzale Giulio Cesare,11, Bari | Comitato Etico Dell'aou Consorziale Policlinico Di Bari  Piazzale Giulio Cesare, 11  70124 Bari (Ba) |
| 19 | U.O.C Oculistica, Azienda Ospedaliera San Gerardo, ASST Monza  Via Pergolesi, 33, Monza | Comitato etico della brianza c/o ospedale s. Gerardo Di Monza - Ospedale San  Gerardo  Via Pergolesi 33  20900 Monza (Mb) |
| 20 | S.C. Clinica Oculistica, Ospedale Santa Maria della Misericordia - Loc. S. Andrea delle Fratte, Azienda Ospedaliera di Perugia  Piazzale Giorgio Menghini, 1, Perugia | Comitato Etico Delle Aziende Sanitarie Dell'umbria - Segreteria Scientificoamministrativa  Del Ceas Umbria  Via Mario Angeloni, 61 C/O Regione Umbria  06124 Perugia (Pg) |
| 21 | U.O. di Oculistica, Presidio di Desenzano del Garda, ASST del Garda  Localita' Montecroce, Desenzano del Garda | Comitato Etico Della Provincia Di Brescia C/O Ao Spedali Civili  Piazzale Spedali Civili, 1  25123 Brescia (Bs) |
| 22 | U.O. di Oculistica, Università degli Studi, Azienda Ospedaliera S. Paolo, ASST Santi Paolo e Carlo  Via Antonio di Rudinì, 8, Milano | Comitato Etico Interaziendale Milano Area 1 C/O Asst Sacco Fatebenefratelli  Via Giovanni Battista Grassi, 74  20157 Milano (Mi) |
| 23 | U.O.C. di Oculistica, IRCCS Casa Sollievo della Sofferenza  Viale Cappuccini, 1, San Giovanni Rotondo | Comitato Etico Dell'irccs Istituto Tumori Giovanni Paolo Ii Di Bari - Sezione  Presso La Fondazione Casa Sollievo Della Sofferenza Di San Giovanni  Rotondo  Viale Cappuccini  71013 San Giovanni Rotondo (Fg) |
| 25 | Clinica Oculistica, Ospedale S. Giovanni di Dio, Azienda Ospedaliero-Universitaria di Cagliari  Via Ospedale, 46/54, Cagliari | Comitato Etico Indipendente Dell'azienda Ospedaliera Universitaria Di  Cagliari  Via Ospedale, 54  09124 Cagliari (Ca) |
| 26 | U.O.C. di Oculistica, Ente Ecclesiastico Ospedale Generale Regionale F. Miulli  Strada Prov. 127 Acquaviva - Santeramo Km. 4, 100, Acquaviva delle Fonti | Comitato Etico Dell'aou Consorziale Policlinico Di Bari  Piazzale Giulio Cesare, 11  70124 Bari (Ba) |
| 27 | S.O.D. Clinica Oculistica, Università degli Studi, A.O.U. Osp. Riuniti Umberto I-GM Lancisi-G. Salesi  Via Conca, 71 – Località Torrette, Ancona | Comitato Etico Regionale (Cer) Delle Marche C/O Azienda Ospedaliera  Universitaria Ospedali Riuniti Umberto I-Lancisi-Gm Salesi Di Ancona  Via Conca  60126 Ancona (An) |
| 29 | U.O.C. di Oculistica, Presidio Ospedaliero di Milazzo - ASP di Messina  Contrada Villaggio Grazia, Milazzo | Comitato Etico Interaziendale Della Provincia Di Messina C/O Aou  Policlinico g. Martino  Via Consolare Valeria, 24  98124 Messina (Me) |
