# Supplementary material for: Effectiveness of anti-vascular endothelial growth factors in neovascular age-related macular degeneration and variables associated with visual acuity outcomes: Results from the EAGLE study
Source: PLoS One. 2021 Sep 1;16(9):e0256461. doi: 10.1371/journal.pone.0256461 (PMC8409622; doi:10.1371/journal.pone.0256461)
Supplement: S2 Table — (DOCX) [file pone.0256461.s006.docx]

**Table S2:** **Demographics and baseline ocular and disease characteristics in OE population**

| **Parameters** | **OE Population**  (N=745) |
| --- | --- |
| Mean (SD) age, years | 75.6 (8.8) |
| Gender, Female, n (%) | 412 (55.3) |
| Race, Caucasian, n (%) | 739 (99.19) |
| **Time from diagnosis to treatment (days)** | |
| n | 690 (92.62) |
| Median | 15 |
| Q1; Q3 | 6; 34 |
| **MNV types (treated eye), n (%)** | |
| Classic (type II) | 214 (28.72) |
| Mixed | 59 (7.92) |
| ND | 135 (18.12) |
| Occult (type I) | 251 (33.69) |
| PCV | 36 (4.83) |
| RAP | 50 (6.71) |
| OE population: all enrolled patients who had at least one anti-VEGF injection;  MNV, macular neovascularization; n, number of patients; ND, not determined; OE,overall exposed; PCV; polypoidal choroidal vasculopathy; RAP, retinal angiomatous proliferation; SD, standard deviation | |
