# Supplementary material for: Effectiveness of anti-vascular endothelial growth factors in neovascular age-related macular degeneration and variables associated with visual acuity outcomes: Results from the EAGLE study
Source: PLoS One. 2021 Sep 1;16(9):e0256461. doi: 10.1371/journal.pone.0256461 (PMC8409622; doi:10.1371/journal.pone.0256461)
Supplement: S3 Table. Median (annualized) anti-VEGF injections in OE and EA study population — (DOCX) [file pone.0256461.s007.docx]

**Table S3: Median (annualized) anti-VEGF injections in OE and EA study population**

| **Statistical parameters** | **OE population** | | | **EA population** | | |
| --- | --- | --- | --- | --- | --- | --- |
|  | **Year 1** | **Year 2** | **Overall** | **Year 1** | **Year 2** | **Overall** |
| **n** | 745 | 610 | 745 | 617 | 524 | 617 |
| **Median (injections)** | 5 | 2.4 | 7.9 | 5 | 2.7 | 8 |
| **Min; Max** | 1; 20.6 | 0; 32.7 | 1; 36.7 | 1; 20.6 | 0; 32.7 | 1; 36.7 |
| **Q1;Q3** | 4; 7 | 0; 4.6 | 5; 11 | 4; 7 | 0; 5 | 5; 11 |
| EA, effectiveness analysis, OE, overall exposed;Q, quartile; VEGF, vascular endothelial growth factor. | | | | | | |
