# Supplementary material for: Effectiveness of anti-vascular endothelial growth factors in neovascular age-related macular degeneration and variables associated with visual acuity outcomes: Results from the EAGLE study
Source: PLoS One. 2021 Sep 1;16(9):e0256461. doi: 10.1371/journal.pone.0256461 (PMC8409622; doi:10.1371/journal.pone.0256461)
Supplement: S4 Table — (DOCX) [file pone.0256461.s008.docx]

**Table S4: Summary statistics of VA (ETDRS letters) and change from baseline in ETDRS at 1 year and 2 years (EA population) as per available measures**

| **Parameters** | **Mean (SD) baseline VA (ETDRS)** | **Mean (SD) VA at**  **year 1** | **Change in ETDRS at year 1** | **Mean (SD)**  **VA at**  **year 2** | **Change in ETDRS at year 2** | **P-value** |
| --- | --- | --- | --- | --- | --- | --- |
| EA  (n=617) | 53.43 (22.8) | NA | | | | |
| 1stCA _EA  (n=429) | 56.13 (21.22) | 58.58 (22.25) | 2.45 (19.36) | NA | NA | 0.0005 |
| 2ndCA _EA (n=335) | 57.23 (20.99) | NA | NA | 55.89 (23.3) | −1.34 (20.85) | 0.3984 |
| **P*-value refers to the Mann Whitney test for $H_{0}$: $\Delta$=0, where $\Delta$ is the change in ETDRS from baseline. Of note the test was computed directly on the difference, therefore is not a paired test  ETDRS, early treatment diabetic retinopathy study; 1stCA _EA, first year completer Effectiveness Analysis set; 2ndCA _EA, second year completer Effectiveness Analysis set; SD, standard deviation | | | | | | |
