# Supplementary material for: Effectiveness of anti-vascular endothelial growth factors in neovascular age-related macular degeneration and variables associated with visual acuity outcomes: Results from the EAGLE study
Source: PLoS One. 2021 Sep 1;16(9):e0256461. doi: 10.1371/journal.pone.0256461 (PMC8409622; doi:10.1371/journal.pone.0256461)
Supplement: S5 Table — (DOCX) [file pone.0256461.s009.docx]

**Table S5: List of EAGLE study investigators and affiliation**

| **S.No.** | **Principal Investigator** | **Affiliation** |
| --- | --- | --- |
| 1 | Iaculli Cristiana | S.C. di Oculistica, Università degli Studi, Presidio Ospedali Riuniti di Foggia, Az. Osp. Ospedali-Università OO.RR. Foggia *Via Luigi Pinto, Foggia* |
| 2 | Vadalà Maria | U.O.C. di Oculistica, Università degli Studi di Palermo, Az. Ospedaliera Universitaria Policlinico P. Giaccone  *Via Liborio Giuffrè, 13, Palermo* |
| 3 | Fenicia Vito | U.O.D. Oculistica, Università La Sapienza, Azienda Ospedaliera Sant'Andrea  *Via di Grottarossa, 1035/1039, Roma* |
| 4 | Vujosevic Stela | S.C.O. di Oculistica, Azienda Ospedaliero-Universitaria Maggiore della Carità  *Corso Mazzini, 18, Novara* |
| 5 | Massimo Nicolò | Clinica Oculistica – DINOGMI, Ospedale Policlinico San Martino IRCCS  *Largo Rosanna Benzi, 10, Genova* |
| 6 | Nardi Marco | U.O. Oculistica Universitaria, Presidio Ospedaliero di Cisanello, Az. Ospedaliero Universitaria Pisana  *Via Paradisa, 2, Pisa* |
| 7 | Ricci Federico | U.O.S.D. Patologie Retiniche Unità Patologie Oculari Croniche Degenerative, Università degli Studi Tor Vergata, Fondazione Policlinico Tor Vergata  *Viale Oxford, 81, Roma* |
| 8 | Lanzetta Paolo | S.O.C. Clinica Oculistica, Università degli Studi, Presidio Ospedaliero Santa Maria della Misericordia di Udine, Azienda Sanitaria Universitaria Integrata di Udine  *P.le S.M. della Misericordia, 15, Udine* |
| 9 | Reibaldi Michele | Divisione di Oftalmologia, Università degli Studi, Presidio Ospedaliero Gaspare Rodolico, Az. Osp. Univ. Policlinico-Vittorio Emanuele  *Via Santa Sofia, 78, Catania* |
| 10 | Mastropasqua Leonardo | Clinica Oftalmologica, Università G. D'Annunzio, P.O. Ospedale Clinicizzato SS. Annunziata Colle Dell'Ara, ASL n. 2 - Lanciano Vasto Chieti  *Via dei Vestini, 5, Chieti* |
| 11 | Virgili Gianni | S.O.D. Oculistica - Ottica Fisiopatologica, Università degli Studi di Firenze, Azienda Ospedaliero-Universitaria Careggi  *Largo Giovanni Alessandro Brambilla, 3, Firenze* |
| 12 | Staurenghi Giovanni | U.O.C di Oculistica, Università degli Studi, Azienda Ospedaliera Luigi Sacco Polo Universitario, ASST Fatebenefratelli Sacco  *Via Giovanni Battista Grassi, 74, Milano* |
| 13 | Bandello Francesco | U.O. di Oculistica, IRCCS Ospedale San Raffaele  *Via Olgettina, 60, Milano* |
| 14 | Viola Francesco | U.O.C. di Oculistica Fondazione IRCCS Ca' Granda Ospedale Maggiore Policlinico, Università degli Studi,Via Manfredo Fanti, 6, Milano |
| 15 | Boscia Francesco | U.O. di Oftalmologia Clinica Oculistica, Azienda Ospedaliero-Universitaria di Sassari  *Viale San Pietro, 43, Sassari* |
| 16 | Giustolisi Rosalia | U.O.S. Centro Maculopatie Clinica Oculistica, Università La Sapienza, A.O. Policlinico Umberto I  *Viale del Policlinico, 155, Roma* |
| 17 | Varano Monica | U.O.S. Retina Medica, Presidio Ospedaliero Britannico, IRCCS Fondazione G.B. Bietti  Via di Santo Stefano Rotondo, Roma |
| 18 | Furino Claudio | U.O. Oftalmologia Universitaria, Università degli Studi, Az. Osp. Univ. Consorziale Policlinico di Bari  *Piazzale Giulio Cesare,11, Bari* |
| 19 | Coppola Michele | U.O.C Oculistica, Azienda Ospedaliera San Gerardo, ASST Monza  *Via Pergolesi, 33, Monza* |
| 20 | Cagini Carlo | S.C. Clinica Oculistica, Ospedale Santa Maria della Misericordia - Loc. S. Andrea delle Fratte, Azienda Ospedaliera di Perugia  *Piazzale Giorgio Menghini, 1, Perugia* |
| 21 | Pucci Vincenzo | U.O. di Oculistica, Presidio di Desenzano del Garda, ASST del Garda  *Localita' Montecroce, Desenzano del Garda* |
| 22 | Migliavacca Luca | U.O. di Oculistica, Università degli Studi, Azienda Ospedaliera S. Paolo, ASST Santi Paolo e Carlo  *Via Antonio di Rudinì, 8, Milano* |
| 23 | Laborante Antonio | U.O.C. di Oculistica, IRCCS Casa Sollievo della Sofferenza  *Viale Cappuccini, 1, San Giovanni Rotondo* |
| 25 | Peiretti Enrico | Clinica Oculistica, Ospedale S. Giovanni di Dio, Azienda Ospedaliero-Universitaria di Cagliari  *Via Ospedale, 46/54, Cagliari* |
| 26 | Micelli Ferrari Tommaso | U.O.C. di Oculistica, Ente Ecclesiastico Ospedale Generale Regionale F. Miulli  *Strada Prov. 127 Acquaviva - Santeramo Km. 4, 100, Acquaviva delle Fonti* |
| 27 | Mariotti Cesare | S.O.D. Clinica Oculistica, Università degli Studi, A.O.U. Osp. Riuniti Umberto I-GM Lancisi-G. Salesi  *Via Conca, 71 – Località Torrette, Ancona* |
| 29 | Romeo Giuseppe | U.O.C. di Oculistica, Presidio Ospedaliero di Milazzo - ASP di Messina  *Contrada Villaggio Grazia, Milazzo* |
