# Supplementary material for: Effectiveness of anti-vascular endothelial growth factors in neovascular age-related macular degeneration and variables associated with visual acuity outcomes: Results from the EAGLE study
Source: PLoS One. 2021 Sep 1;16(9):e0256461. doi: 10.1371/journal.pone.0256461 (PMC8409622; doi:10.1371/journal.pone.0256461)
Supplement: S1 Appendix — (DOCX) [file pone.0256461.s010.docx]

**S1 Appendix:**

**Statistical analysis**

Quantitative variables were described by means, standard deviation (SD), median, first and third quartile, minimum and maximum, as appropriate depending on the distribution’s shape, while categorical variables by absolute and relative frequency. The bilateral 95% CI was given where relevant. The statistical primary unit of the analyses was the “first treated eye” for each patient. Descriptive statistics analysis of baseline and demographic characteristics and nAMD diagnosis and ocular history disease in the Overall Exposed (OE), Effectiveness Analysis (EA) set populations and Excluded patients (from the EA) are provided. The OE population set was defined as all enrolled patients who received at least one dose of anti-VEGF treatment during the enrollment period. The EA population set included all patients in the OE who had a baseline and at least a post-baseline VA assessment. The EA population versus excluded patients were compared using a t-test or Mann-Whitney test for continuous variables, depending on the distribution’s shape, and Chi-square test for categorical variables.
